# Intraportal *versus* Systemic Pentoxifylline Infusion after Normothermic Liver Ischemia: Effects on Regional Blood Flow Redistribution and Hepatic Ischemia-Reperfusion Injury

**DOI:** 10.1155/2013/689835

**Published:** 2013-08-29

**Authors:** Edson A. Ribeiro, Luiz F. Poli-de-Figueiredo, Rodrigo Vincenzi, Flavio H. F. Galvao, Nelson Margarido, Mauricio Rocha-e-Silva, Ruy J. Cruz

**Affiliations:** ^1^Research Division, Heart Institute (InCor), University of Sao Paulo School of Medicine, 05403-900 Sao Paulo, SP, Brazil; ^2^Hospital do Cancer de Muriae, Fundacao Cristiano Varella, 36880-000 Muriae, MG, Brazil; ^3^Liver Transplant Unit, University of Sao Paulo School of Medicine, Avenida Dr Arnaldo 455, Suite 3206, 01246-903 Sao Paulo, SP, Brazil

## Abstract

Pentoxifylline (PTX) has been shown to have beneficial effects on microcirculatory blood flow. In this study we evaluate the potential hemodynamic and metabolic benefits of PTX during hepatic ischemia. We also test the hypothesis that portal PTX infusion can minimize the I/R injury when compared to systemic infusion. *Methods*. Twenty-four dogs (18.1 ± 0.7 kg) were subjected to portal triad occlusion (PTO) for 45 min. The animals were assigned to 3 groups: CT (control, PTO, *n* = 8), PTX-syst (PTO + 25 mg/Kg of PTX IV, *n* = 8), and PTX-pv (PTO + 25 mg/Kg of PTX in the portal vein, *n* = 8). Animals were followed for 120 min. Systemic hemodynamics, gastrointestinal tract perfusion, oxygen-derived variables, and liver enzymes were evaluated throughout the experiment. *Results.* Animals treated with PTX presented significantly higher CO in the first hour after reperfusion, when compared to the CT (*~*3.7 vs. 2.1 L/min, *P* < 0.05). Alanine aminotransferase (ALT) was similar in the PTX groups two hours after reperfusion but significantly higher in the CT (227 vs. *~*64 U/L, *P* < 0.05). *Conclusion*. PTX infusion was associated with hemodynamic benefits and was able to minimize liver injury during normothermic hepatic I/R. However, local PTX infusion was not associated with any significant advantage over systemic route.

## 1. Introduction

Despite technical advances in liver surgery in the last decades, the consequences of liver ischemia/reperfusion injury remain a major concern for surgeons. Liver ischemia/reperfusion (I/R) injury is a complex cascade of events mediated by numerous inflammatory cells and molecular mediators, resulting in hepatocyte death and systemic inflammatory response. The degree of inflammatory response and organ dysfunction is dependent on duration of liver ischemia and underlying liver disease. In this setting, activation of hepatic macrophages plays an important role. Macrophages have been responsible for the release of various inflammatory mediators, including but not limited to tumor necrosis factor alpha (TNF-*α*). Several studies have shown that the inhibition of TNF-*α* production or its neutralization after isolated hepatic I/R decreases polymorphonuclear neutrophil infiltration with further reduction of the I/R injury [1–3]. 

Pentoxifylline (PTX) is a methylxanthine derivative that displays vasodilatory effects on peripheral blood vessels, particularly on the liver [4–8]. In addition, PTX has other important pharmacological properties that may be responsible for the minimization of hepatic I/R including; attenuation of leukocyte-endothelial interactions, reduction of blood viscosity, and suppression of cytokine release by the overstimulated Kupffer cells [9–13]. 

Previously, we and others have shown the cardiovascular benefits of PTX infusion in a canine model of hemorrhagic shock [10, 14]. However, no existing study has ever examined the effects of PTX infusion on systemic and locoregional hemodynamics in a large animal model of hepatic I/R. With that in mind, we have designed this study to evaluate the potential hemodynamic and metabolic benefits of PTX infusion during normothermic hepatic ischemia. We have also tested the hypothesis that regional PTX infusion (i.e., portal) can minimize the I/R injury when compared to systemic infusion. 

## 2. Methods

The experimental protocol was approved by the Institutional Review Board, in adherence with the “Principles of Laboratory Animal Care” formulated by the National Society for Medical Research and the “Guide for the Care and Use of Animals” by the National Institutes of Health.

### 2.1. Animal Preparation

Twenty-four male mongrel dogs, weighing 17.9 ± 0.7 kg, were fasted for 12 hours prior to the study, with free access to water. Anesthesia was induced with an intravenous injection of 0.1 mg/kg of morphine sulfate followed by 25 mg/kg of pentobarbital sodium. Additional doses of pentobarbital, 2 mg/kg, were used as necessary. A cuffed endotracheal tube was placed to allow mechanical ventilation with a 1.0 fraction of oxygen inspired, at a tidal volume of 15 mL/kg (670 Takaoka, ventilator, São Paulo, SP, Brazil). The respiratory rate was adjusted to maintain an initial arterial pCO_2_ at 40 ± 5 mmHg. A urinary bladder catheter was placed for urinary drainage. During surgical preparation, a heating pad was used to maintain normothermia. The animals received lactated Ringer solution, 10 mL/kg/h, to compensate for fluid losses.

A polyethylene cannula (P240) was placed in the right carotid artery to measure mean arterial pressure and to collect arterial blood samples for blood gas, pH, bicarbonate, base deficit, hematocrite and hemoglobin analyses. A 7.5 Fr flow-directed thermodilution fiberoptic pulmonary artery catheter with thermal filament (CCOmbo 744H7.5F, Edwards Swan-Ganz, Baxter Edwards Critical Care, Irvine, CA, USA) was introduced through the right external jugular vein with its tip placed in the pulmonary artery, guided by the pressure wave tracings. This catheter was connected to a cardiac computer (Vigilance, Baxter Edwards Critical Care, Irvine, CA, USA) to measure cardiac output using 3-mL bolus injections of isotonic saline at 20°C every fifteen minutes. The same catheter was also used to collect mixed venous blood samples for gas analysis. All pressure-measuring catheters were connected to disposable pressure transducers (Transpac Transducer, Abbott, Chicago, IL, USA) and then to a Biopac Data Acquisition System (Model MP100, Biopac Systems, Goleta, CA, USA) for continuous recording of systemic and pulmonary artery pressures.

The abdomen was opened through a median celiotomy. The gastroduodenal and right gastric arteries were isolated and ligated. The portal vein and common hepatic artery were dissected, and transit time ultrasonic flow probes were placed around these vessels and connected to a flowmeter (T206 Transonic Volume Flowmeter, Transonic Systems, Inc, Ithaca, NY, USA). A fluid-filled polyethylene catheter was placed in the portal vein, through the pancreatoduodenal vein, to collect blood samples. 

During hepatic ischemia, a portosystemic venous-venous bypass was used to decompress the splanchnic bed. Initially, a splenectomy was performed, and a polyethylene cannula was placed in the splenic vein for continuous drainage of the splanchnic territory. Another polyethylene cannula was introduced in the left femoral vein for venous drainage. The tube was primed with heparin-containing saline solution and connected to a centrifugal pump. Pump flow was adjusted to maintain the baseline portal vein blood flow. Immediately before reperfusion, mannitol 20% (50 mL) and calcium gluconate (400 mg) were injected intravenously, and a continuous infusion of dopamine (5 *μ*g/Kg/min) was also initiated.

### 2.2. Experimental Design and Measured Variables

After completion of the surgical preparation, 45 minutes were allowed for stabilization and baseline measurements (BL) readings. Prior to performing Pringle's maneuver the animals were randomly assigned in three experimental groups: CT (control, portal triad clamping, *n* = 8), PTX-syst (portal triad clamping + 25 mg/Kg of PTX intravenous systemically, *n* = 8), and PTX-pv (portal triad clamping + 25 mg/Kg of PTX in the portal vein, *n* = 8). Pringle's maneuver was performed with a noncrushing vascular clamp for 45 minutes (P45). During the ischemic period all animals received an infusion of 500 cc of normal saline solution (NS) with or without PTX in the portal vein. All solutions were infused using an automatic electronic pump. After hepatic pedicle declamping, the animals were then observed for additional 120 minutes (R0 to R120), and then euthanized with an overdose of pentobarbital and potassium chloride. 

Mean systemic and pulmonary arterial pressures (MAP and MPAP, resp.) and hepatic artery and portal vein blood flows (HABF and PVBF, resp.) were continuously recorded. Cardiac output was determined every 15 minutes, using 3-mL bolus injections of isotonic saline at 20°C. Each determination was the arithmetic mean of three consecutive measurements when their differences did not exceed 10%. Arterial, portal, and mixed venous base deficits, pH, pCO_2_, pO_2_, oxygen saturation, hemoglobin, bicarbonate levels, and ALT and DHL were measured at baseline (BL), at the end of hepatic ischemia (P45) and 15, 60, and 120 after reperfusion (R15, R60, and R120, resp.). All blood samples were analyzed, immediately after their collection, by a Stat Profile Ultra Analyzer (Nova Biomedical, Waltham, MA, USA). Systemic and splanchnic oxygen delivery, consumption, and extraction (DO_2_syst, VO_2_syst, O_2_ERsyst, DO_2_splanch, VO_2_splanch, and O_2_ERsplanch, resp.) were calculated using standard formulae. 

### 2.3. Statistical Methodology

Results are presented as mean ± standard error of mean. Statistical analysis was performed using a Statistic Package for Social Sciences for Windows software (version 10.0, SPSS, Chicago, IL). Differences between groups were analyzed using repeated measure analysis of variance and post hoc Tukey's test. Statistical significance was considered for *P* values less than 0.05.

## 3. Results

### 3.1. Systemic Hemodynamic Parameters

Portal triad occlusion promoted a slight reduction in MAP in all animals (Table 1). During early reperfusion (R60) lower MAP levels were observed in all three groups in comparison to baseline measurements. However, there was no significant difference between groups. At the end of experiment the MAP in both PTX-sys and PTX-pv presented a partial recovery (Table 1). Mean pulmonary arterial pressure remained stable throughout the experiment, with no differences between groups.

**Figure 1:**
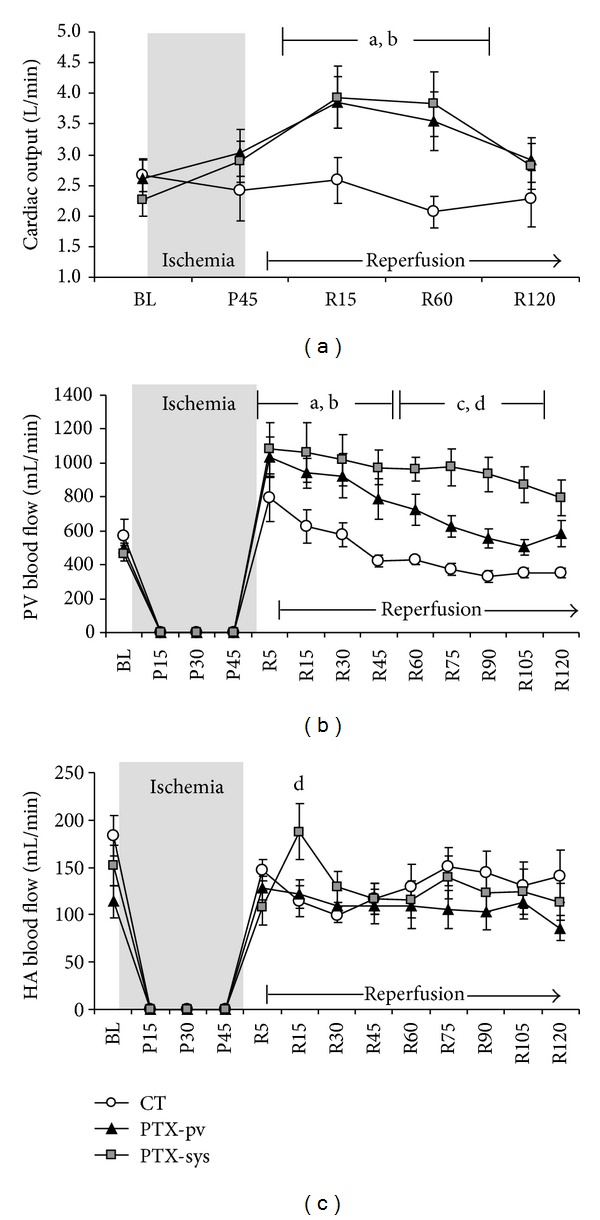
(a) Cardiac output (L/min), (b) and (c) portal vein and hepatic artery blood flows (mL/min) during the experimental protocol. The animals were randomly assigned into three groups: CT (control, portal triad clamping, *n* = 8), PTX-syst (portal triad clamping + 25 mg/Kg of PTX intravenous systemically, *n* = 8), and PTX-pv (portal triad clamping + 25 mg/Kg of PTX in the portal vein, *n* = 8). BL: baseline; P45: 45 min after Pringle's maneuver; R15, R60, and R120: 15, 60, and 120 min after reperfusion. Data are shown as mean ± SEM. ^a^Indicates *P* < 0.05 for PTX-pv and PTX-sys *versus* BL; ^b^bindicates *P* < 0.05 for PTX-pv and PTX-sys versus CT; ^c^indicates *P* < 0.05 for PTX-sys versus BL; ^d^indicates *P* < 0.05 for PTX-sys versus CT.

Baseline cardiac output was similar in all three groups. However, animals treated with PTX presented significantly higher CO in the first hour after reperfusion, when compared to the control group (Figure 1(a)). Cardiac output was similar to baseline levels in all three groups by the end of the experiment (R120). After portal vein declamping, a significant acidosis was observed without any difference between groups throughout the reperfusion period. 

### 3.2. Hepatic Blood Flow

Systemic and intraportal infusionS of PTX promoted a significant improvement in portal vein blood flow during reperfusion when compared to CT animals. In all groups a rapid but not sustained restoration of PVBF was observed after portal triad declamping. However, animals in the control group demonstrated a progressive reduction in portal blood flow during the reperfusion phase when compared to PTX-treated animals. Moreover, 30 minutes after reperfusion, until the end of the observation, animals which received systemic PTX presented significantly higher PVBF when compared with baseline values and to the CT group (Figure 1(b)).

All animals presented a sustained recovery of hepatic artery blood flow during reperfusion, with no differences between groups (Figure 1(c)). Fifteen minutes after reperfusion, animals treated with systemic PTX presented higher hepatic artery blood flows when compared with the other two groups, but no differences were observed afterwards (Figure 1(c)). In Table 2, we can observe the significant decrease of systemic and splanchnic oxygen delivery in CT group. On the other hand, animals treated with PTX did not present significant changes on DO_2_. A compensatory increase of systemic and regional oxygen extraction was observed in CT group (Table 2).

### 3.3. Serum ALT and LDH Levels

**Figure 2:**
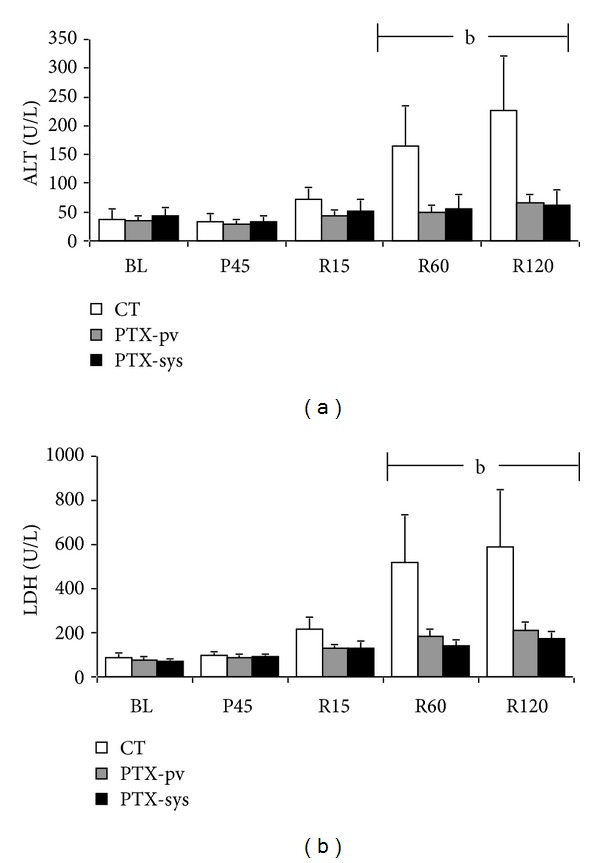
(a) Alanine transaminase (ALT, U/L) and (b) lactate dehydrogenase (LDH, U/L) during the experimental protocol. BL: baseline; P45: 45 min after Pringle's maneuver; R15, R60, and R120: 15, 60, and 120 min after reperfusion. Groups are the same as in Figure 1. Data are shown as mean ± SEM.  ^b^Indicates *P* < 0.05 for CT versus PTX-pv and PTX-sys.

Pentoxifylline exerted a protective effect against ischemia/reperfusion injury. Serum markers of liver injury remained stable throughout the experiment in animals treated with both systemic PTX and intraportal PTX. However, a progressive increase of serum ALT and LDH was observed in CT group after reperfusion (Figures 2(a) and 2(b)).

## 4. Discussion

This study confirms that intravenous systemic infusion of PTX is an effective strategy to prevent liver damage after normothermic I/R. Locoregional PTX infusion (*i.e.,* portal vein) also has demonstrated a protective effect on liver cells, but no advantage was obtained over standard systemic infusion. PTX has been studied in hemorrhagic shock models in combination with different strategies of fluid resuscitation. Besides the beneficial hemodynamic and metabolic effects, we and others have demonstrated that PTX infusion reduces the circulation of inflammatory cytokines, bacterial translocation, and polymorphonuclear neutrophil-endothelial interactions, thus attenuating the systemic inflammatory response syndrome triggered by trauma and hemorrhage [10, 11]. 

In this study, no differences between the groups were observed regarding systemic arterial pressure and pulmonary arterial pressure. However, during reperfusion cardiac output was significantly higher in animals treated with PTX. The precise mechanism responsible for the improvement in cardiac function observed after PTX administration remains unknown. A combination of factors may have contributed to this beneficial effect of PTX on CO, including, but not limited to: (1) the improvement in systemic blood flow secondary to pentoxifylline's ability to enhance red blood cell deformability, leading to vasodilatation and decreased afterload, and (2) the downregulation of tumor necrosis factor-*α* synthesis, which is a potent myocardial depressor released after I/R injury [10, 14, 15]. Interestingly, systemic delivery of pentoxiphylline improved portal blood flow better than intraportal infusion. PTX is extensively metabolized by the liver and erythrocytes to 3-carboxypropyl and 5-hydroxyhexyl metabolites, respectively. We believe that systemic infusion could have circumvented the first-pass metabolism avoiding the extensive PTX biotransformation, explaining the better regional hemodynamic effect with systemic PTX infusion. 

Plasma ALT, AST, and LDH levels are widely used as markers of liver cell damage in animal models and in the clinical setting. In this study, infusion of PTX promoted a protective effect in the liver against I/R injury, with no differences between the two routes of administration. On the other hand, animals that did not receive PTX demonstrated a progressive increase in ALT and LDH levels during reperfusion. 

Downregulation of TNF-*α*, a potent proinflammatory cytokine, is probably one of the most important effects of PTX in preventing liver damage. It has been shown that PTX can inhibit the activation of liver macrophages (Kupffer cells) after an ischemic insult, reducing the production and release of TNF-*α*, and consequently other inflammatory mediators, resulting in less end-organ injury [15–17]. 

The short period of observation is an important limitation of our study. Because of our study design, we could not correlate the data presented herein with mortality or development of multiple organ dysfunction, issues that must be addressed in a future study. Also, the use of a venous by-pass is not routine in liver surgery; however, we decided to use an extrahepatic shunt during portal triad clamping to avoid the deleterious effects of intestinal congestion. Small bowel congestion can lead to intestinal mucosal injury, bacterial translocation, and systemic inflammatory response affecting the hemodynamic and metabolic responses during and after normothermic isolated liver I/R [18–20]. We have previously shown that active spleno-femoral shunt maintains the systemic hemodynamic stability, with an effective decompression of splanchnic bed during portal triad occlusion. The deleterious hemodynamic and metabolic effects observed during the reperfusion period, such as transitory hypotension and acidemia, was mainly associated with the isolated hepatic I/R injury, not with the blood congestion in splanchnic bed [19]. 

We believe that systemic PTX infusion right after reperfusion during liver transplantation could be a useful strategy to improve portal vein blood flow and minimize the deleterious effects of I/R injury. However further investigation is required, in order to evaluate safety and the potential benefits of continuous PTX infusion in short- and long-term graft function and survival.

Despite these limitations, we were able to demonstrate the systemic and hepatosplanchnic hemodynamic benefits of PTX infusion during normothermic hepatic ischemia/reperfusion. We also have shown that pentoxifylline administration could minimize liver damage. However, local PTX infusion was not associated with any significant advantage over systemic route. 

## Figures and Tables

**Table 1:** Mean arterial and pulmonary artery pressures (MAP and MPAP, mmHg), arterial hemoglobin (g/dL), arterial pH in CT (control, portal triad clamping, *n* = 8), PTX-syst (portal triad clamping + 25 mg/Kg of PTX intravenous systemically, *n* = 8), and PTX-pv (portal triad clamping + 25 mg/Kg of PTX in the portal vein, *n* = 8) groups.

	Group	BL	P45	R15	R60	R120
MAP mmHg	CT	100.5 ± 20.4	91.0 ± 21.5	83.3 ± 25.2	67.5 ± 26.4^a,b,c^	73.5 ± 29.4^a^
	PTX-syst	115.3 ± 17.7	85.0 ± 22.2	94.1 ± 21.5	80.2 ± 18.3^a^	87.0 ± 31.2
	PTX-pv	111.6 ± 21.4	84.0 ± 14.8	89.2 ± 20.6	78.0 ± 14.7^a^	87.2 ± 19.2

MPAP mmHg	CT	15.6 ± 2.2	16.8 ± 2.8	16.2 ± 3.9	15.0 ± 4.2	13.0 ± 3.6
	PTX-syst	14.7 ± 3.7	14.6 ± 5.0	16.1 ± 3.2	14.0 ± 2.6	13.7 ± 2.8
	PTX-pv	15.6 ± 4.8	16.5 ± 5.2	17.5 ± 5.7	16.4 ± 5.1	15.7 ± 4.9

Hemoglobin g/dL	CT	12.9 ± 2.1	10.2 ± 2.4	10.5 ± 2.8	11.4 ± 3.1	11.4 ± 3.2
	PTX-syst	13.6 ± 1.7	11.2 ± 2.0	11.1 ± 2.2	10.4 ± 2.2	10.3 ± 2.7
	PTX-pv	12.9 ± 1.7	10.4 ± 1.7	10.4 ± 1.2	10.1 ± 1.5	9.6 ± 1.4

Arterial pH	CT	7.36 ± 0.06	7.30 ± 0.07	7.23 ± 0.05^a^	7.21 ± 0.07^a^	7.21 ± 0.05^a^
	PTX-syst	7.41 ± 0.06	7.32 ± 0.08	7.25 ± 0.09^a^	7.24 ± 0.11^a^	7.24 ± 0.11^a^
	PTX-pv	7.36 ± 0.07	7.28 ± 0.06	7.22 ± 0.07^a^	7.24 ± 0.09^a^	7.24 ± 0.08^a^

Legends: BL: baseline; P45: 45 min after Pringle's maneuver; R15, R60, and R120: 15, 60, and 120 min after reperfusion. Data are shown as mean ± SEM. ^a^Indicates P < 0.05 versus BL; ^b^indicates P < 0.05 versus PTX-sys; ^c^indicates P < 0.05 versus PTX-pv.

**Table 2:** Systemic (syst) and splanchnic (splanc) oxygen delivery, consumption and extraction (DO_2_, VO_2_, and O_2_ ER, resp.) in CT (control, portal triad clamping, *n* = 8), PTX-syst (portal triad clamping + 25 mg/Kg of PTX intravenous systemically, *n* = 8), and PTX-pv (portal triad clamping + 25 mg/Kg of PTX in the portal vein, *n* = 8) groups.

	Group	BL	P45	R15	R60	R120
DO_2_ syst mL/min	CT	483 ± 76	449 ± 58	367 ± 40^a,b,c^	254 ± 29^a,b,c^	211 ± 61^a,b^
	PTX-syst	454 ± 39	496 ± 62	580 ± 76	479 ± 62	381 ± 56
	PTX-pv	411 ± 89	530 ± 43	707 ± 109^a^	578 ± 83^a^	416 ± 63

VO_2 _syst mL/min	CT	10 ± 3.1	12.8 ± 7.8	4.7 ± 0.8	6.9 ± 0.5	7.5 ± 2.1
	PTX-syst	8.5 ± 2	8.5 ± 2.1	8.1 ± 0.6	7.7 ± 1.2	8.8 ± 1.8
	PTX-pv	6.8 ± 0.3	7.7 ± 1.6	10.5 ± 2.9	6.5 ± 1.1	8.4 ± 1

O_2_ ERsyst%	CT	16.9 ± 2.9	17.7 ± 3.9	14.8 ± 3.5	23.7 ± 1.8^a,b^	33.9 ± 2.4^a,b,c^
	PTX-syst	16.7 ± 1.7	16.5 ± 3.4	13.7 ± 1.9	18 ± 3.4	19.9 ± 2.4
	PTX-pv	15.3 ± 1.5	14.2 ± 2.2	18.8 ± 4.5	14 ± 20	20.6 ± 2.9^a^

DO_2_ splanc mL/min	CT	103 ± 28^c^	—	85 ± 55	71 ± 48	54 ± 39^a,c^
	PTX-syst	95 ± 5	—	150 ± 20	93 ± 12	69 ± 7^a^
	PTX-pv	80 ± 16	—	156 ± 31	137 ± 11	111 ± 20

VO_2_ splanc mL/min	CT	6.4 ± 0.4	—	8.2 ± 1.5	9.4 ± 0.8	10 ± 1.1
	PTX-syst	10 ± 1.4	—	17.7 ± 3.4	8.3 ± 2.2	8.7 ± 0.5
	PTX-pv	9 ± 0.9	—	14.5 ± 2.7	10.9 ± 1.7	8.3 ± 1.8

O_2_ ERsplanc%	CT	80 ± 16^b^	—	124 ± 35	156 ± 13^a,b,c^	192 ± 13^a,b,c^
	PTX-syst	121 ± 5	—	108 ± 12	92 ± 4	134 ± 4
	PTX-pv	104 ± 1.3	—	115 ± 36	82 ± 13	87 ± 17

Legends: BL: baseline; P45: 45 min after Pringle's maneuver; R15, R60, and R120: 15, 60, and 120 min after reperfusion. Data are shown as mean ± SEM. ^a^Indicates P < 0.05 versus BL; ^b^indicates P < 0.05 versus PTX-sys; ^c^indicates P < 0.05 versus PTX-pv.
